# The Role of Endoplasmic Reticulum Stress in Cell Injury Induced by Methimazole on Pancreatic Cells

**DOI:** 10.34172/apb.2023.042

**Published:** 2022-01-09

**Authors:** Özge Yazıcı, Mehtap Kara, Tuğçe Boran, Gul Ozhan

**Affiliations:** Department of Pharmaceutical Toxicology, Faculty of Pharmacy, Istanbul University, 34116, Beyazıt, Istanbul, Turkey.

**Keywords:** Methimazole, Drug induced pancreatitis, Toxicity, Reactive oxygen species

## Abstract

***Purpose:*** Methimazole is an anti-thyroid agent, especially as main therapy option for Graves’ disease in children and adults. Drug induced pancreatitis is one of the known adverse effect of methimazole mentioned in case reports. However, the detailed molecular mechanisms of methimazole-induced pancreatitis are still unclear. In this study, the aim is to investigate the adverse effect of methimazole on pancreas cell stress mechanism and apoptosis.

***Methods:*** Cytotoxicity was evaluated in human pancreas/duct (PANC-1) cell line. Total oxidant (TOS) and antioxidant status (TAS) for oxidative stress index, glutathione (GSH) level and endoplasmic reticulum (ER) stress biomarkers were evaluated by ELISA. Reactive oxygen species (ROS) levels and apoptosis were evaluated by flow-cytometer.

***Results:*** The 30% inhibition rate concentration (IC_30_) value was determined as 53 mM in PANC1 cells. The exposure concentrations were in the range of 0-40 mM for 48 hours. Methimazole might induce cellular stress conditions. ROS production increases depending on concentration, and this increase shows parallelism with the increase in ER stress biomarkers such as TOS, ERN1 and CASPASE12. Conversely, there was no significant difference between control and exposure groups in terms of apoptosis.

***Conclusion:*** In conclusion, methimazole might have triggered the mechanisms of inflammation or autophagy in the pancreatic cells. However, there is still a need for *in vitro* and *in vivo* studies including other cellular parameters related to apoptosis.

## Introduction

 Methimazole, carbimazole (prodrug of methimazole) and propylthiouracil are three thionamide compounds used in hyperthyroidism therapy.^1^ Methimazole is one of the main therapy option for Graves’ disease. Thus, methimazole is one of the options that could be safely use in both adults and children.^2^ Adverse effects such as hypersensitivity, decrease in the number of leukocyte, agranulocytosis, gastrointestinal symptoms and hepatic dysfunction can occur in patients with methimazole treatment. Furthermore, the patients with acute pancreatitis induced methimazole reported abdominal pain, increased pancreatic enzyme levels and imaging findings like pancreas swelling in case-reports.^3-6^ Acute pancreatitis was added to the list of adverse effects of methimazole based on six case reports in 2019; also, methimazole product information was changed as including acute pancreatitis with European Medicine Agency (EMA) warning.^1^

 Drug induced acute pancreatitis occurs rarely; however, it could be life threatening.^7^ One of the main molecular mechanism underlying acute pancreatitis is oxidative stress. The increased cellular reactive oxygen species (ROS) causes the death of pancreatic cells.^8^ It has been reported that cellular calcium level changes, mitochondrial membrane permeability changes, endoplasmic reticulum (ER) stress, unfolded protein response (UPR), apoptosis and autophagy are main underlying molecular mechanisms of acute pancreatitis pathogenesis. Pancreatic cells are under high risk of ER stress due to its higher protein synthesis capacity. During the ER stress in acinar cells, the UPR system is activated to restore cellular homeostasis.

 The three functional pathways of get involved with three main pathways such as ERN1, activating transcription factor 6 (ATF6) and protein kinase RNA-like ER kinase (PERK) pathways. Glucose-regulated protein 78 (Grp78), Grp90 and heat shock protein 90 (Hsp90) are the main regulator of UPR signaling. Growth arrest- and DNA damage-inducible gene 153 (DDIT3) is the downstream effector protein regulating gene expression in the cell during UPR signaling.^9^ ERN1 induction in the pancreas cells for different reasons may cause cell death.^10^ One of the key factor for severity of pancreatitis is the increased pancreatic cell death. caspase 12, one of the important cytoplasmic caspase, forms a link between ER stress and apoptosis. When caspase 12 activates UPR signaling via ER stress, it in turn activates important caspase 9 and caspase 3 resulting in apoptosis.^11,12^

 The underlying molecular mechanism of methimazole induced acute pancreatitis has been not clarified yet. Thus, in this study, we aimed to observe toxic effect mechanism of methimazole in human pancreas duct epithelioid carcinoma cells, by assessing the cellular cytotoxicity, oxidative stress, ER stress and apoptosis. PANC-1 cells have optimal cell proliferation, gene expression and adhesion profiles to study on pancreas functions and these cell types were used in many studies.^13-16^

## Materials and Methods

 Methimazole was purchased from Chemie Uetikon GmbH (Lahr, Germany). Human pancreas/duct (PANC-1) cell line (CRL-1469) was obtained from the American Type Culture Collection (ATCC, Virginia, USA). ELISA kits for total antioxidant status (TAS), total oxidant status (TOS), ERN1, DDIT3, Grp78, Hsp90, and caspase12 from Elabscience Biotechnology Co. Ltd (Texas, USA). Glutathione (GSH) Elisa Kit was obtained from YH Biosearch laboratory (Shanghai, China). FITC Annexin V Apoptosis Detection Kit with propidium iodide (PI) was from BioLegend (CA, USA). The cell culture chemicals and all other cell culture supplements were from Multicell Wisent (Quebec, Canada).

 The PANC-1 cells were nourished in Dulbecco’s modified eagle medium: nutrient mixture F-12 (DMEM F-12) media include 10% fetal bovine serum (FBS) and 100 U/100 μg/mL penicillin/streptomycin at 37°C in a humidified incubator with 5% CO_2_. For cytotoxicity assessment, the methimazole concentrations were in the range of 0-200 mM for 48h exposure. The medium was used for control. After 48 hours exposure, the optical densities (ODs) were measured at 590 nm by a microplate spectrophotometer reader (Epoch, Germany), and IC_30_ was calculated.

 Considering IC_30_ value, the cells were exposed to 20, 30 and 40 mM methimazole for 48 hours with control group for oxidative stress, ER stress and apoptosis evaluation. All assay was done in triplicates and performed in three independent days.

 The cellular ROS generation was assessed by 2ʹ,7ʹ-dichlorodihydrofluorescein diacetate (H_2_DCF-DA) assay with flow-cytometer.^17,18^ The cells (1 × 10^5^) were seeded in 6-well plates, and incubated for 24 hours. The fluorescence intensities were measured with ACEA NovoCyte flow-cytometer (San Diego, CA, USA) at 488 nm. The results were expressed as the percentage of median fluorescence intensity. The GSH, TAS and TOS levels were measured by Elisa kits according to manufacturer’s instructions. The GSH levels were expressed as μmol/10^4^ cell. The TAS and TOS levels were expressed as U/mL. The ERN1, DDIT3, Grp78, Hsp90 and caspase12 levels were assessed with Elisa kits according to the manufacturer’s instructions. The results were expressed as ng/mL. Annexin V Apoptosis Detection Kit with PI was used to investigate the apoptosis/necrosis pattern of the cells by flow-cytometer ACEA NovoCyte flow-cytometer at 488 nm according to manufacturer’s instructions. The results were expressed as the percent of the total cell amount.

 For the statistically analysis, H_2_DCF-DA fluorescence were detected from the areas on the FSC/SSC graph that obtained from the cells and analysis was performed with the NovoExpress software computer program. The presence of free oxygen radicals was expressed as a percentage of the total cell amount (M2%). TAS, TOS, ERN1, DDIT3, Grp78, Hsp90 and caspase 12 results statistical analysis were performed with one-way ANOVA post hoc Dunnett’s *t* test by using SPSS version 20.0 (SPSS Inc., Chicago, Illinois, USA), and the results were given as mean ± standard deviation (SD). *P* ≤ 0.05 was the level of significance.

## Results and Discussion

**Figure 1:**
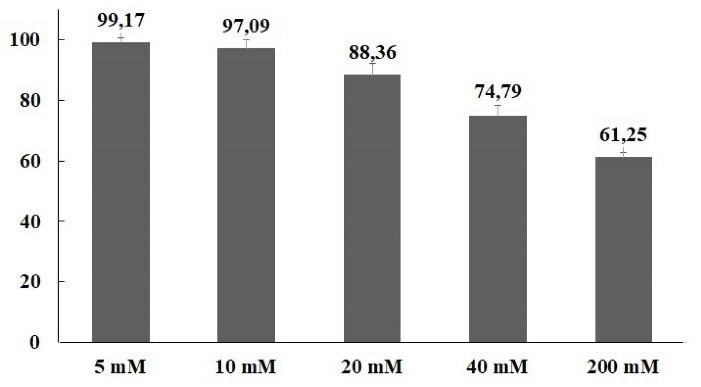
Cell viability inhibition effects of methimazole in PANC-1 cells according to MTT results for 48 h exposure. The cell viability decreased methimazole concentration dependent

 Acute pancreatitis’ annual incidence has a range of 4.9-73.4 cases/100 000 people and the mortality rate is 1.5-4.2% in epidemiological studies. This rate increases up to 30% depending on the severity of pancreatitis.^19,20^ It is important to determine the etiological causes of acute pancreatitis for appropriate treatment and follow-up. The most common causes of acute pancreatitis are biliary, alcohol, hypertriglyceridemia, hypercalcemia, drug-related, autoimmune, hereditary/genetic and anatomical anomalies.^21^ The annual incidence of drug-induced acute pancreatitis is 0.1-5%. More than 500 drugs triggering acute pancreatitis were listed by World Health Organization (WHO) and the number is rising in each passing year. The mechanisms of drug-induced acute pancreatitis are generally based on case reports, case-control studies, animal studies, and other experimental data. The potential mechanisms for drug-induced acute pancreatitis include pancreatic duct narrowing, cytotoxic and metabolic effects, accumulation of toxic metabolites or mediators, and hypersensitivity reactions. However, the mechanism of drug induced acute pancreatitis has not been clarified yet. Various methods have been proposed to assess the causality in drug-induced acute pancreatitis. Currently, the drug-induced pancreatitis classification system is widely used for uniform assessment of causality in pancreatoxicity.^22-27^ There are limited methimazole induced acute pancreatitis case reports in the literature.^4-6,28-30^ However, this is the first study to evaluate oxidative and ER stress mechanisms role in methimazole induced acute pancreatitis The cytotoxic effect of methimazole on the hepatocytes was evaluated by Heidari et al,^31^ isolated from male Sprague-Dawley rats, for 2 hours exposure in the range of 1-15 mM. A concentration-dependent cytotoxic effect was detected in hepatocytes isolated from rats with trypan blue assessment and the IC_50 _value was found as 10 mM. In the present study, it was observed that methimazole decreased the cell viability depending on concentration with 48h exposure in PANC-1 cells. IC_30 _value of methimazole was 53 mM (Figure 1).

**Figure 2:**
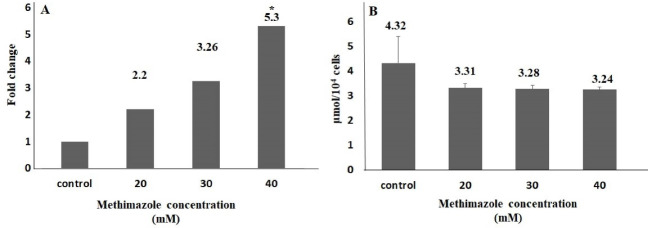
ROS production and GSH level in methimazole treated PANC-1 cells for 48h. ROS production level significantly increased in 40 mM group compared to control (A). There was no significant difference for GSH parameters between control and exposure groups (B) (*P < 0.05)

**Figure 3:**
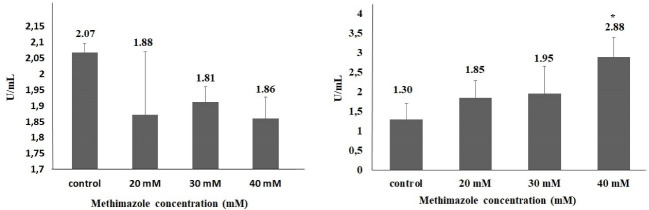
TAS (A) and TOS (B) levels in methimazole treated PANC-1 cells for 48 h (*P < 0.05)

 One of the important mechanism that play a key role in the pathogenesis of acute pancreatitis is oxidative stress-related cell death. The severity of pancreatitis is related with oxidant-antioxidant balance in the early phase of disease.^8,32^ In the study,methimazole increased ROS production levels in all concentrations compared to control group; however, the value of significance was seen only in 40-mM concentration. The rate of ROS production increase in 20, 30 and 40 mM concentrations were 2.2, 3.3 and 5.3 fold, respectively, and there was a significant difference only in 40-mM concentration (*P* < 0.05). However, the TOS levels increased significantly at only 40 mM (2.2-fold;*P* < 0.05). On the other hand, there was no significant difference as compared with control group in terms of the GSH and TAS levels (Figures 2 and 3).

 It has been reported that the producing reactive metabolites play important role in methimazole induced liver injury. Heidari et al^31^ reported that methimazole caused a significant decrease in GSH levels in hepatocytes isolated from Sprague-Dawley male rats (*P* < 0.05). In addition, methimazole increased ROS levels 1.8-fold compared to the control group at 10 mM (*P* < 0.05). In another study,^33^ methimazole induced serious liver damage and GSH depletion induced significantly (2.5-fold) at 200 mg/kg in Swiss albino mouse (*P* < 0.05). Vickers et al^34^ compared 8 drugs, including methimazole, in an *ex vivo* 3D multicellular human liver section model. They observed that methimazole increased GSH levels by 15%-37% at 24 hours, 11%-49% at 48 hours and 30%-49% at 72 hours at 500 μM. Kobayashi et al^35^ found there was a 1.5-fold significant decrease in the amount of GSH in the hepatic tissue in BALB/c female mice 6 hours after exposure to 450 mg/kg oral methimazole. Niknahad et al^36^ observed a significant increase in ROS levels in the liver mitochondria isolated after exposure to ≤ 400 mg/kg methimazole in BALB/c male mice (*P* < 0.001). In vivo evaluation revealed that the amount of mitochondrial ROS was higher compared to control animals (*P* < 0.001). In our study, GSH amounts were 3.31 and 3.24 μmol per 10^4^ cells at 20 and 40 mM concentrations in PANC-1 cell lines (*P* > 0.05). A significant increase (5.3-fold) was found in the ROS level at 40 mM (*P* < 0.05) compared to control group.

**Figure 4:**
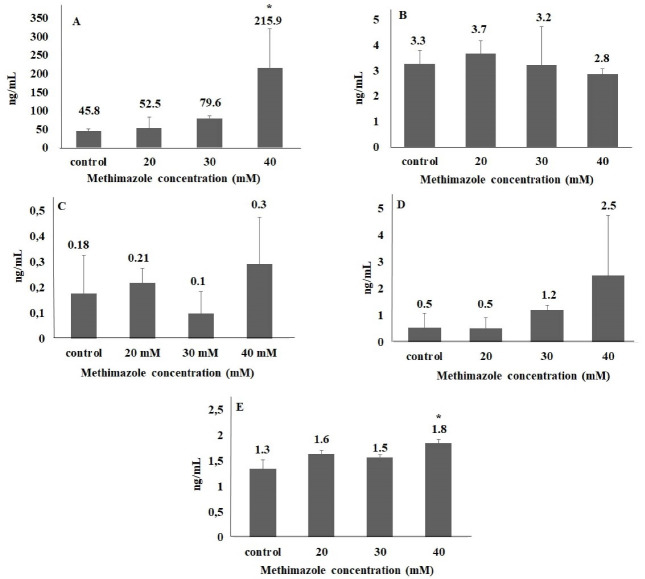
ER stress parameters in methimazole treated PANC-1 cells for 48h. (A) ERN1, (B) DDIT3, (C) HSP90, (D) Grp78, (E ) Caspase 12 (*P < 0.05)

**Figure 5:**
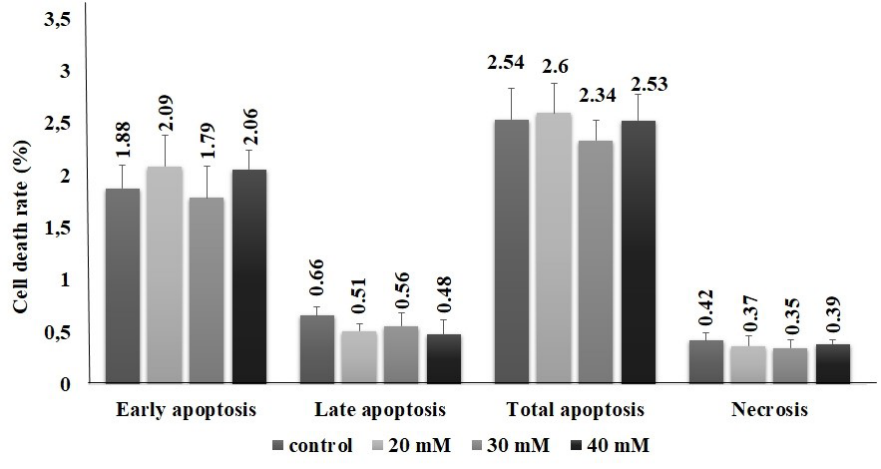
Apoptosis/necrosis inducing potential of methimazole in PANC-1 cells for 48 h. The assay was performed with Annexin V with PI by flow-cytometer. There was no significant difference between control and exposure groups for all cell death types as early, late apoptosis and necrosis. The results were presented as percentage of the total cell amount

 In the present study, no significant difference was found between groups in parameters of DDIT3, Hsp90 and Grp78. In 40 mM group, ERN1 and caspase 12 levels significantly increased in the order of 4.7- and 1.4-fold, respectively. We suggest that methimazole may induce ER stress pathway in pancreas cells via ERN1 pathway (Figure 4). Although ROS level and ER stress parameters increased in 40-mM concentration methimazole group in PANC-1 cells, apoptosis was not induced. Moreover, there was no significant difference between the groups for apoptosis parameter (Figure 5). Drug-induced ER stress not only triggers apoptosis mechanism but also induces cellular mechanisms of inflammation and autophagy.^37^ This is the first study to evaluate methimazole treatment effects on ROS, ER stress and apoptosis in pancreas cells. According to the results of our study, methimazole induces ER stress mechanism via ERN1 pathway that could be associated with pancreatitis. However, cell death was not induced. Given that the changes in autophagy and inflammation markers were not examined in this study, the last step leading to pancreatitis in pancreatic cells could not be clarified.

## Conclusion

 In this study, we evaluated the toxicity of methimazole in PANC-1 cell line through oxidative stress and endoplasmic reticulum stress parameters. According to cell viability study result, the IC30 value of methimazole was calculated as 53 mM for 48 hours. ROS, TOS, ERN1 and CASPASE12, cellular stress pathways biomarkers, levels increased with methimazole. However, it was observed that although cellular stress markers increased, apoptosis was not induced in cells. These results suggest that the effect of methimazole on the pancreas is not mediated by induction of apoptosis. In summary, the data obtained from our study, in which basic evaluations were made for elucidating the mechanisms underlying the risk of methimazole induced acute pancreatitis, need to be supported by further *in vitro* and *in vivo* studies that determine the relationship between methimazole and its metabolites with the risk of pancreatitis.

## Acknowledgments

 The present work was supported by the Research Fund of Istanbul University (Project No. 36298).

## Author Contributions

**Conceptualization:** Özge Yazıcı, Mehtap Kara, Tuğçe Boran, Gül Özhan.

**Data curation:** Özge Yazıcı, Mehtap Kara, Tuğçe Boran.

**Formal Analysis: **Özge Yazıcı.

**Funding acquisition: **Gül Özhan.

**Investigation: **Özge Yazıcı, Gül Özhan.

**Methodology:** Özge Yazıcı, Tuğçe Boran.

**Project administration:** Özge Yazıcı, Gül Özhan.

**Resources:** Özge Yazıcı.

**Software:** Özge Yazıcı, Tuğçe Boran.

**Supervision: **Tuğçe Boran, Gül Özhan.

**Validation: **Özge Yazıcı, Tuğçe Boran.

**Visualization:** Özge Yazıcı, Mehtap Kara, Tuğçe Boran.

**Writing – original draft:** Özge Yazıcı, MMehtap Kara, Tuğçe Boran.

**Writing – review & editing:** Özge Yazıcı, Mehtap Kara, Tuğçe Boran, Gül Özhan.

## Ethical Issues

 Not applicable.

## Conflict of Interest

 The authors declare that there are no financial competing interests.
